# Exosomes derived from human umbilical cord mesenchymal stem cells inhibit hepatocyte pyroptosis via miR-423-5p/ZBP1 in acute liver failure

**DOI:** 10.1007/s13577-025-01248-1

**Published:** 2025-07-04

**Authors:** Dan Xie, Lina Yu, Ziyang Wang, Gongqin Qiu, Shi Ouyang

**Affiliations:** 1 Guangzhou Key Laboratory of Enhanced Recovery After Abdominal Surgery, Department of Infectious and Liver Disease, The Fifth Affiliated Hospital of Guangzhou Medical University, Guangzhou, 510515 China; 2 School of Public Health, North China University of Science and Technology, Tangshan, 063210 China; 3 School of Medicine, Hunan University of Medicine, Huaihua, 418099 China

**Keywords:** Exosomes, miR-423-5p, hucMSCs, Acute liver failure, Pyroptosis

## Abstract

Human umbilical cord mesenchymal stromal cells (hucMSCs) have emerged as a promising therapeutic option for acute liver failure (ALF). However, the detailed mechanism by which hucMSCs modulate hepatocyte pyroptosis in ALF remains unclear. In this study, we induced ALF in mice using acetaminophen (APAP). Mice were intravenously injected with 1 × 10^6^ hucMSCs/ hucMSCs-Exo via the tail vein 6 h after APAP administration. Liver pathological changes were assessed by hematoxylin and eosin (H&E) staining. Subsequently, an in vitro model of liver cell failure and pyroptosis model was established using LO2 cells treated with lipopolysaccharide (LPS) and adenosine 5’-triphosphate (ATP). The levels of cell pyroptosis marker caspase-1 were detected by flow cytometry analysis, RT-qPCR assay, and western blot assay. The study employed a comprehensive approach, including flow cytometry analysis, RT-qPCR assay, miRNA sequencing, and luciferase reporter gene experiments. hucMSCs and hucMSCs- exosomes (MSCs-Exo) inhibited cell inflammation to improve ALF in vivo model of ALF and inhibited hepatocyte pyroptosis in LO2 cells induced by ATP and LPS. MiR-423-5p emerged as a potential mediator of the anti-pyroptotic effects of hucMSCs-Exo, with ZBP1 identified as one of its downstream targets. Subsequent validation confirmed that miR-423-5p targets ZBP1 to regulate pyroptosis. These findings highlight the role of hucMSCs-derived exosomal miR-423-5p in inhibiting hepatocyte pyroptosis in LO2 cells induced by ATP and LPS. miR-423-5p serves as a crucial mediator in this process, targeting ZBP1, a protein significantly involved in the pyroptotic pathway. Our findings may provide basics for further research on mechanism of hucMSCs and present a promising cell-free strategy treating ALF.

**Supplementary Information:**

The online version contains supplementary material available at 10.1007/s13577-025-01248-1.

## Introduction

Acute liver failure (ALF), also referred to as fulminant hepatic failure, is a severe and often life-threatening clinical condition marked by sudden and extensive hepatocyte damage [1], resulting in a swift decline in liver function and the development of complications that contribute to a high mortality rate and an unfavorable prognosis. Consequently, liver transplantation emerges as the primary treatment for ALF [2] and is regarded as the paramount life-saving measure [3]. Globally, liver transplantation is recognized globally as a critical intervention, with 1-year survival rates reaching 79% in Europe and 84% in the United States [4]. However, the limited availability of donor organs frequently hinders prompt transplantation, underscoring the imperative for alternative therapies aimed at repairing liver injury [5]. This necessity is crucial not only to alleviate donor deficits but also to provide viable options for doctors and patients facing an ALF diagnosis.

Recent studies have highlighted the role of macrophage polarization and hepatocyte pyroptosis in the pathogenesis of ALF, suggesting that controlling the balance between pro-inflammatory M1 and anti-inflammatory M2 macrophages, as well as inhibiting pyroptosis, can help reduce liver injury [6]. Additionally, our previous work has shown that Z-DNA-binding protein 1(ZBP1) promotes hepatocyte pyroptosis in acute liver injury by regulating the PGAM5/ROS pathway, further emphasizing the importance of targeting pyroptosis in ALF [7]. Recent studies have revealed that, alongside necrosis, pyroptosis—an immune-cell-based programmed cell death—plays a central role in the inflammatory responses contributing to the pathogenesis of ALF [8]. Typically, pyroptosis is activated by the protease caspase-1 at the inflammasome NLRP3 (nucleotide-binding oligomerization domain-like receptor protein 3). Upon activation, caspase-1 cleaves interleukin 1 beta (IL-1β) and IL-18 to produce mature cytokines, regulating their secretion and thereby facilitating pyroptosis [9]. Moreover, activated caspase-1 can cleave Gasdermin-D (GSDMD) at inflammasomes, producing N-terminal GSDMD fragments that form membrane pores, ultimately leading to cell death [10, 11]. As a result of the release of cellular contents and inflammatory cytokines, a cascade of pro-inflammatory cytokines ensues, promoting pyroptosis [12]. Pyroptosis is a newly discovered type of programmed cell death that distinctively differs from other types of which, what was previously believed to be the protagonist of related pathogenesis. Dependent on caspase-1 activation [13], it eventually forms pores in the cell membrane, left the cell susceptible to propidium iodide (PI) staining, a method to detect cells with damaged membranes. Such membranous pores also result in cell swelling distinguishable under optical microscopy [14]. Importantly, the previous studies have indicated that inhibiting pyroptosis has a hepatoprotective effect and anti-inflammatory function in mouse models of ALF induced by lipopolysaccharide (LPS)/D-galactosamine (D-GAIN) [15]. Similarly, targeting the inhibition of hepatocyte pyroptosis in human subjects holds promise as a potentially effective treatment strategy for patients with ALF.

Mesenchymal stromal cells (MSCs) have emerged as a promising therapeutic option for ALF. Human umbilical cord mesenchymal stromal cells (hucMSCs) exhibit the ability to modulate both innate and adaptive immune functions and have displayed potential in the treatment of various inflammatory diseases [16, 17]. Previous studies revealed that hucMSCs transplantation can effectively improve liver function and promote hepatocyte proliferation following ALF [18], since after transplantation, hucMSCs is reported to migrate toward injured tissues, inhibiting inflammation reactions, and enhancing liver cell proliferation [19]. Additionally, hucMSCs have demonstrated anti-pyroptotic effects in various contexts, including breast cancer cells, cardiomyocytes, and microglia [20–22]. Research has highlighted the anti-pyroptotic impact of hucMSCs in ALF mice through the secretion of interleukin-10 (IL-10). However, the precise mechanisms underlying the inhibitory effects of hucMSCs on hepatocyte pyroptosis remain unclear.

Accumulating evidence suggests that hucMSCs can effectively ameliorate ALF through paracrine mechanisms, with recent research focusing on exosomes for more detailed insights [23, 24]. Exosomes are nano-sized vesicles with a diameter of 30–150 nm, and can be secreted by different cells [25, 26]. As such, exosomes are essential mediators of cell-to-cell communication by transferring various biomolecules, including proteins, lipids, and nucleic acids [mRNAs, microRNA (miRNA) and DNA, etc.] [27, 28]. Notably, exosomes derived from hucMSCs (hucMSCs-Exos) have been shown to reduce NLR Family Pyrin Domain-Containing 3 (NLRP3) inflammasome expression, downstream inflammatory factors, Alanine Aminotransferase (ALT), and Aspartate Aminotransferase (AST) levels in an ALF mouse model [29]. Meanwhile, hucMSCs-Exos, by inhibiting pyroptosis, exhibits benefit in the treatments of numerous inflammation-induced diseases, including colitis, intervertebral disk degeneration (IVDD), and myocardium injury [30–32]. Despite indications of the potential of exosomes derived from human umbilical cord mesenchymal stromal cells (hucMSCs-Exos) in treating ALF involving the regulation of inflammasome-induced pyroptosis, the molecular mechanisms remain incompletely understood.

In this study, we used LO2 cells, human liver cells, to establish an in vitro model of hepatocyte pyroptosis. These cells are widely used in liver research due to their hepatocyte-like characteristics and their capability to simulate liver cell responses under various experimental conditions. This aims of this study is to investigate the significant therapeutic role of hucMSCs-Exos in ALF through their anti-pyroptosis effect and explore the genes and pathways implicated in this process using microRNA sequencing (miRNA-Seq). First, our study investigates whether hucMSCs can improve the pathological and cell pyroptosis phenotypes in ALF. Subsequently, we explore the influence of hucMSCs on liver cell pyroptosis in the context of ALF and investigate the anti-pyroptosis effects of exosomes derived from hucMSCs, assessing their potential in ALF treatment. It is crucial to determine whether miR-423-5p is a key molecule in regulating liver cell pyroptosis in hucMSCs-Exo. Based on the research findings of hucMSCs-Exo, we discuss the potential of cell-free therapeutic approaches in the treatment of ALF. 

## Materials and methods

### Isolation, culture, and identification of hucMSCs

The isolation and culture methods of hucMSCs used in this study are based on previously published protocols [33, 34]. HucMSCs involved in this experiment were obtained from the umbilical cords of healthy infants with full consent from their well-informed delivery women. The criteria for healthy infants: (1) full-term birth (gestational age greater than or equal to 37 weeks); (2) Absence of major congenital diseases or health issues at birth; (3) No genetic or chromosomal abnormalities; (4) No signs or symptoms of infectious diseases; (5) Normal growth parameters, such as weight, height, and head circumference within normal ranges. After harvesting, the hucMSCs were placed in sterile sodium heparin tubes (25 U sodium heparin per mL) for 4 h. Under strict aseptic conditions, the cord was cut longitudinally along the lumen of the umbilical vein, the intima of the umbilical vein was peeled off with toothed forceps, with the Wharton’s Jelly covering the artery slightly peeled off, and the two arteries withdrew in a smooth manner. The remaining part was mainly Wharton’s Jelly, which was cut into small pieces of about 1 mm and placed into a T175cm^2^ tissue culture flasks (Corning, USA) using a medicine spoon and then incubated in a humidified atmosphere containing 95% air and 5% CO2. Any movement of the flasks was strictly prohibited for the first 7 days, and afterward, 10 mL of complete medium was added to the culture flasks, while the tissue blocks were removed after the hucMSCs had been cultivated. Morphological features of hucMSCs were examined by optical microscopy. The immunophenotype (CD90, CD29, CD44, CD73, CD105, CD34, and CD45) of hucMSCs was analyzed by flow cytometer. All hucMSCs involved in this study were used for further experiments. In this study, all participants were reviewed and approved by the Ethics committee of The Fifth Affiliated Hospital of Guangzhou Medical University (KY01-2021-09-09), and each pregnant woman, from whom the hucMSCs were isolated, provided their written informed consent of participation.

### Isolation and identification of exosomes

After hucMSCs reached approximately 80% confluence in serum-free medium (SFM, Gibco, USA), the cell culture supernatant was collected. This supernatant underwent a two-step low-speed centrifugation process (300 r/min for 10 min followed by 2000 r/min for 10 min) to eliminate cell debris. Subsequently, the supernatant is further cleared of larger organelles and intact cells by centrifugation at a high speed of 10000×r/min for 30 min. The supernatant is then subjected to ultracentrifugation at 100000×r/min for 70 min to collect exosomes, after which the supernatant is discarded. Then, the exosome pellet is resuspended in phosphate-buffered saline (PBS) at a pH of 7.4 and subjected to another ultracentrifugation at 100,000 r/min for 70 min to wash the exosomes and remove any potential impurities. Finally, the supernatant is discarded, and the exosome pellet is resuspended in PBS before being stored at −  80 °C to maintain its activity. All centrifugation steps were carried out at 4℃.

Particles from hucMSCs, which were believed to be the exosomes, were resuspended and further diluted in 1 ml PBS to analyze their number and size. The morphologic characteristics of hucMSCs-Exos were observed by transmission electron microscopy (TEM) (Thermo Fisher, Talos F200i S/TEM, Czechia). 5 μl of the above sample was resuspended and dropped onto formvar carbon-coated 200-mesh grids for a 10 min incubation, and was fixed using 2.5% glutaraldehyde for 5 min and stained with 2% uranyl acetate for 1 min. Grids were examined using TEM at 80 kV [35]. Isolated hucMSCs-Exos samples were injected into a nanoparticle-tracking analysis (NTA) (Particle Metrix, ZetaView, Germany) to capture Brownian motion for particle visualization. The Stokes–Einstein equation was applied to measure the average velocity of the particles for the estimation of particle sizes [31]. All human participants involved in these studies have been reviewed and approved by the Ethics Committee of The Fifth Affiliated Hospital of Guangzhou Medical University (KY01-2021-09-09).

### Quantification of exosomes

The concentration of exosomes was precisely measured using a BCA protein assay kit (Beyotime, China). The specific steps are as follows: The exosome pellet was resuspended in PBS and mixed with RIPA lysis buffer (containing PMSF) in equal volume, and then incubated on ice for 30 min with gentle shaking every 10 min. Subsequently, the samples were centrifuged at 12,000×*g* for 15 min at 4 °C, and the supernatant was used for the BCA protein assay. The total protein content of the exosomes was calculated using a standard curve method, which determined the final concentration of the exosomes. In vitro experiments were conducted with a final exosome concentration of 10 μg/ml, a concentration validated in the previous studies to significantly inhibit hepatocyte pyroptosis without causing cytotoxicity.

Establishment of ALF mouse model and groups treatment

The preparation procedures for the ALF mouse model in this study are based on previously published protocols[36, 37]. Mice (C57BL/6 J, aged 8 weeks) were purchased from Shanghai Cyagen Biotechnology Co., Ltd. Four groups were divided randomly, including normal group, ALF group, ALF + hucMSCs group, and ALF + Exo group (*n* = 5 for each group). The first group was the normal group and received no treatment. For the other groups, mice were intraperitoneally injected with acetaminophen (APAP) (300 mg/kg, Sigma-Aldrich, St Louis, MO, USA) to establish a mouse model of APAP-induced ALF [37]. hucMSCs (1 × 10^6^) were administered via tail veil injection. Exo were injected through the tail vein at a dose of 20 mg/kg body weight. This method of subsequent treatment has been validated in the previous studies [8]. The animal experiments were conducted in strict accordance with the guidelines of the Institutional Animal Care and Use Committee of Guangzhou Medical University. The study protocol was reviewed and approved by the IACUC, and the approval number is [N2025-25024].

### Establishment of hepatocyte pyroptosis model

LO2 cells (HL-7702) were purchased from Procell Life Science & Technology Co. Cells and cultured in Dulbecco’s modified Eagle’s medium (DMEM) (GIBCO, USA) containing 10% fetal bovine serum (FBS) (GIBCO, USA) supplemented with 1% (v/v) penicillin/streptomycin (GIBCO, USA) in a humidified atmosphere containing 5% CO2 at 37  ℃. To induce hepatocyte pyroptosis, LO2 cells were stimulated with 100ng/ml Lipopolysaccharides (LPS) (Sigma, USA) for 24 h, followed by 5 mmol/L adenosine triphosphate (ATP) (Abmole, USA) for 30 min (the ATP+LPS treatment group) [38].

### Cell culture and treatment

To prepare the in vitro co-culture models, the ATP + LPS + MSCs’ group was obtained by co-culturing the LO2 cell model group and hucMSCs using a Transwell system with a 0.4 μm pore polyester membrane (Costar Corning, USA). Briefly, 1 × 10^5^/mL LO2 cells were seeded in the lower compartments in 6-well plates for 24 h. Then, the culture medium was replaced by fresh serum-free DMEM medium containing LPS/ATP. Meanwhile, matched Transwell chambers containing 1 × 10^5^/mL hucMSCs were inserted into the 6-well plates and co-cultured with LO2 cells for 24 h. Subsequently, LO2 cells were harvested for following tests.

The exosomes (10 μg/ml) were added to the LO2 cell model group in the ATP+LPS+Exos group. Meanwhile, LO2 cells were set up as normal hepatocytes’ control group. Next, changes in related indicators of pyroptosis were detected and analyzed.

### MiR-423-5p treatment

To investigate the effects of miR-423-5p on hepatocyte pyroptosis, LO2 cells were treated with miR-423-5p mimics or a negative control (NC) using Lipofectamine 2000 (Invitrogen, 11668-027, USA) according to the manufacturer’s protocol. Briefly, 2×10^5^ LO2 cells were seeded into 24-well plates and allowed to adhere overnight. On the following day, the medium was replaced with serum-free DMEM, and miR-423-5p mimics or NC were transfected into the cells at a final concentration of 50 nM. After 48 h of transfection, the cells were treated with ATP (5 mmol/L) and LPS (100 ng/ml) for 30 min to induce pyroptosis. The cells were then harvested for further analysis, including flow cytometry and RT-qPCR assays.

### Western blot analysis

The total protein of exosomes was analyzed by western blotting. HucMSCs-Exos were mixed with RIPA lysate (Leagene, China) together with phenylmethanesulfonyl fluoride (PMSF) (Sigma, USA) in equal volume, and were then placed on ice for 30 min with a shake every 10 min. Then, the samples were centrifuged at 12,000×*g* for 15 min at 4 °C for determining the total protein of the mixture through a BCA protein assay kit (Beyotime, China). Samples were mixed with 5 × SDS-PAGE ladder buffer (Beyotime, China) in a volume ratio of 4:1, boiled at 100 ℃ for 10 min, and underwent centrifugation at 14,000×*g* for 10 min at room temperature. Such prepared samples were subjected to SDS-PAGE, and were then transferred to PVDF membrane (Millipore, USA). Primary antibodies employed were anti-CD9 (Abcam#ab236630, 1:1000, UK) and anti-CD81(Abcam# ab79559, 1:1000, UK). Antibodies were incubated overnight at 4 ℃ and were incubated on the second day with the corresponding secondary antibody (1:2000) for 1 h at RT and were exposed to color with ECL luminescent reagent, with membranes being consequently developed through the appropriate imaging system (Bio-Rad, USA).

To further confirm the activation of caspase-1 and GSDMD, Western blotting was performed to detect their cleaved (active) forms. Total protein was extracted from LO2 cells ATP + LPS, ATP + LPS + hucMSCs, and ATP + LPS + hucMSCs-Exos. The primary antibodies used were anti-cleaved caspase-1 (Asp10) (Cell Signaling Technology, #3866, 1:1000) and anti-cleaved GSDMD (Cell Signaling Technology, #13783, 1:1000). The membranes were incubated with the corresponding secondary antibodies (1:2000) and developed using ECL luminescent reagent.

### Serum ALT and AST activity detection

Mouse blood samples obtained from the retroorbital vein were left to stand at room temperature for 1 h and then centrifuged at 2500 rpm for 15 min. The supernatant was carefully aspirated into EP tubes for further use. The ALT and AST activities in mouse serum were measured using the ALT and AST assay kits from Nanjing Jiancheng Biotechnology Research Institute. The specific procedures were performed according to the detailed steps outlined in the kit instructions.

### Real time-quantitation polymerase chain reaction (RT-qPCR)

Total RNA was extracted from LO2 cells and liver tissues using the TRIzol reagent kit, and the extracted RNA was analyzed for concentration and normalization using a spectrophotometer. Following the instructions of the BioRT reverse transcription amplification kit, RNA was reverse transcribed into cDNA under the following conditions: 42 ℃ for 45 min, 95 ℃ for 5 min, and 4 ℃ for 5 min. Subsequently, the cDNA was subjected to cyclic amplification according to the SYBR Green kit instructions, with amplification conditions as follows: 94 ℃ for 3 min, 94 ℃ for 30 s, and 56.8 ℃ for 45 s, at 72 for 45 s, for a total of 40 cycles. The relative gene expression level was calculated using the 2^^−ΔΔCT^ method, with GAPDH as the reference gene for normalization. The primer sequences are detailed as Table 1.

**Table 1:** The sequences of all primers

Gene name	Forward primer sequence (5ʹ–3ʹ)	Reverse primer sequence (5ʹ–3ʹ)
Caspase 1	TTTCCGCAAGGTTCGATTTTCA	GGCATCTGCGCTCTACCATC
GSDMD	GTGTGTCAACCTGTCTATCAAGG	CATGGCATCGTAGAAGTGGAAG
ZBP1	AACATGCAGCTACAATTCCAGA	AGTCTCGGTTCACATCTTTTGC
GAPDH	GGAGCGAGATCCCTCCAAAAT	GGCTGTTGTCATACTTCTCATGG

### Flow cytometry analysis

FAM FLICA™ caspase-1 (YVAD) Assay kit (AbD Serotec, Germany) was applied according to the manufacturer’s protocol. We seeded an appropriate amount of LO2 cells in a 6-well plate, processed under different conditions for the corresponding time, collected, and washed the cells 2 times with pre-chilled PBS. Then, the cell pellets were resuspended in 300 μL 1 × binding buffer containing caspase-1-FITC for 1 h and incubated for 15 min by adding 5 μL PI. Cells were examined by an Accuri C6 flow cytometer (BD, Biosciences, USA). Results were processed by the FlowJo software. The pyroptotic cell death in hepatocytes was also determined by flow cytometry analysis [14].

### MicroRNA sequencing and data analysis

Exosomal miRNA sequencing was performed by Shanghai Cutseq Biomedical Technology Co. Ltd (Shanghai, China). After filtering, the remaining reads were mapped to the miRbase database (version 22) to quantify their expression against known miRNAs. The read count for each miRNA was obtained by performing comparison and correction. Then, the processed sequences were compared and annotated. MiRNAs with a count number of more than 20 were screened for further mRNA target gene prediction using multiple miRNA prediction tools, including mirRnda, Targetscan, miRDB, mirTarBase, and miRwalk. Functional analysis of target genes was performed by the Gene Ontology (GO) and Kyoto Encyclopedia of Genes and Genomes (KEGG) databases, respectively.

### Luciferase reporter gene assay

The 2×10^5^ LO2 cells were seeded into 24-well plates and co-transfected with pmir-ZBP1 wild-type (WT) plasmids together with miR-423-5p mimics using lipo2000 (Invitrogen, 11668-027, USA). miR-empty vector was used as a control. The cell lysates were then transferred to a 96-well plate after 48h. A Dual-Luciferase Assay Kit (Promega, USA) was used to detect the luciferase activity using a Fluorescence Spectrophotometer F-4500 (Promega, E1910, USA). Final results and illustrative graphs were generated by the GraphPad Prism 6.0 software.

### Hematoxylin and eosin (H&E) staining

Mouse liver tissues are immersed in a 10% neutral formalin solution and fixed for 24 h, and then made into sections approximately 3 mm thick. After a series of processes including gradient dehydration with ethanol, transparency with xylene, paraffin embedding, and sectioning, the sections are cut into thin slices of 4 μm using a microtome. The paraffin strips are divided and placed in warm water at 37 °C to pick up the sections, and then baked on a slide warmer for 5 h. Subsequently, the sections are deparaffinized using xylene, ethanol, and distilled water in sequence, followed by H&E staining. After staining, the sections are covered with neutral balsam for observation of tissue damage under a microscope.

### Statistical analysis

In this study, each experiment was independently repeated at least three times using biological replicates to ensure the reliability and robustness of the data. All experiments included both technical and biological repeats to ensure the validity of the results. All statistical analyses were performed using SPSS 21.0 software. For comparisons between the two groups, we first checked the normality and homogeneity of variance of the data, and then selected appropriate statistical tests based on the characteristics of the data distribution. For normally distributed data, we used Student’s *t* test; for non-normally distributed data, we employed the non-parametric Mann-Whitney *U* test. In cases of multiple comparisons, we applied Bonferroni correction to control the false discovery rate. All data were presented as mean ± standard deviation (SD), and *P* values less than 0.05 were considered statistically significant. We also generated charts in GraphPad Prism 6.0 software to visually display the results and data distribution.

## Results

### HucMSCs protect LPS-induced ALF

**Fig. 1:**
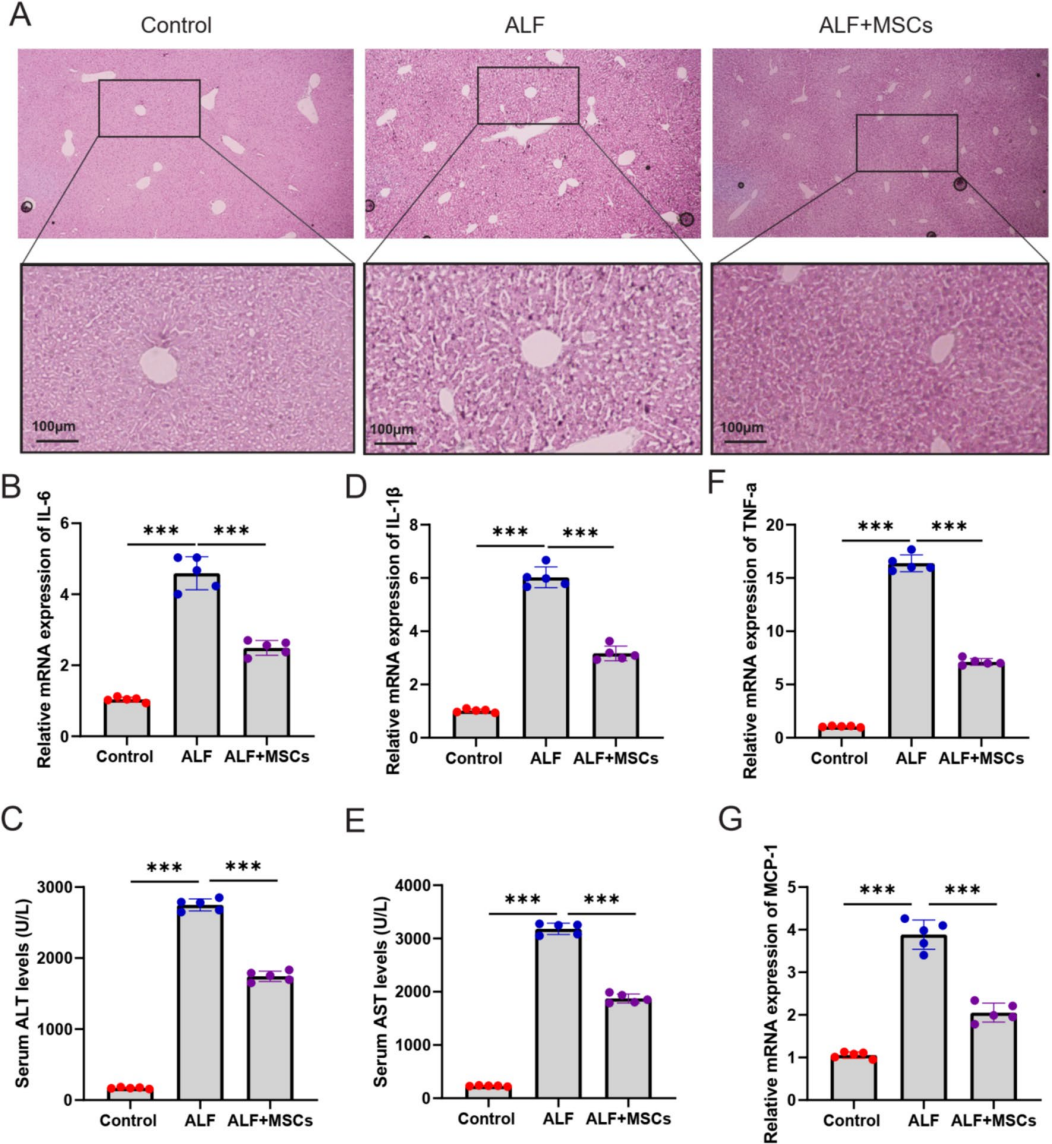
HucMSCs protect LPS-induced acute liver failure (ALF). **A** The liver tissues were collected and fixed for H&E staining. Scale bar: 100 μm. RT-qPCR assay was used to detect the mRNA level of inflammatory mediators IL-1β (**B**), MCP-1 (**C**), TNF-α (**D**), and IL-6 (**E**). Biochemical assays of hepatic indexes: **F** alanine aminotransferase (ALT) and **G** glutamine-oxaloacetate transaminase (AST). *N* = 5 mice per group; the data are represented by five individual experiments. The experiment was performed by biological repeats. *P < 0.05, **P < 0.01, and ***P < 0.001

To explore the effects of hucMSCs on ALF, we first characterized the characters and differentiation potential of hucMSCs. Flow cytometry analysis revealed that the gated hucMSCs exhibited robust expression of classic mesenchymal stromal cell (MSC) surface markers, including CD90, CD29, CD44, CD73, and CD105, with expression levels exceeding 98% for each marker (Supplementary Fig.  1 A). In addition, the hematopoietic stromal cell markers CD34 and CD45 were either negative or minimally expressed, further confirming the purity of the hucMSC population. Additionally, under an optical microscope, the hucMSCs displayed a typical uniform morphology, characterized by spindle-shaped and wheel-shaped arrangements (Supplementary Fig. 1B). These findings collectively indicate that the hucMSCs used in this study are of high purity and possess the characteristic features of MSCs. The ALF model was established in vivo by the administration of APAP. H&E staining was used to assess the pathological changes. After APAP injection, centrilobular necrotic foci, cell apoptosis, and inflammatory cells’ infiltration were observed in the liver. After treatment with MSCs, centrilobular necrosis, cell apoptosis, and inflammation were significantly reduced in liver tissues (Fig. 1A). Next, the serum levels of inflammatory factors were assessed. The results showed that most inflammatory factors (IL-1β, MCP-1, TNF-α, and IL-6) were significantly increased after ALF models groups. hucMSCs reduced the levels of inflammatory factors (IL-1β, MCP-1, TNF-α, and IL-6) (Fig. 1B–E). Biochemical assays of hepatic indexes showed a significant improvement in liver damage after hucMSCs’ treatment, including ALT (Fig. 1F) and AST levels (Fig. 1G). Thus, hucMSCs inhibited the secretion of inflammatory cytokines to improve ALF.

### HucMSCs inhibit hepatocyte pyroptosis

**Fig. 2:**
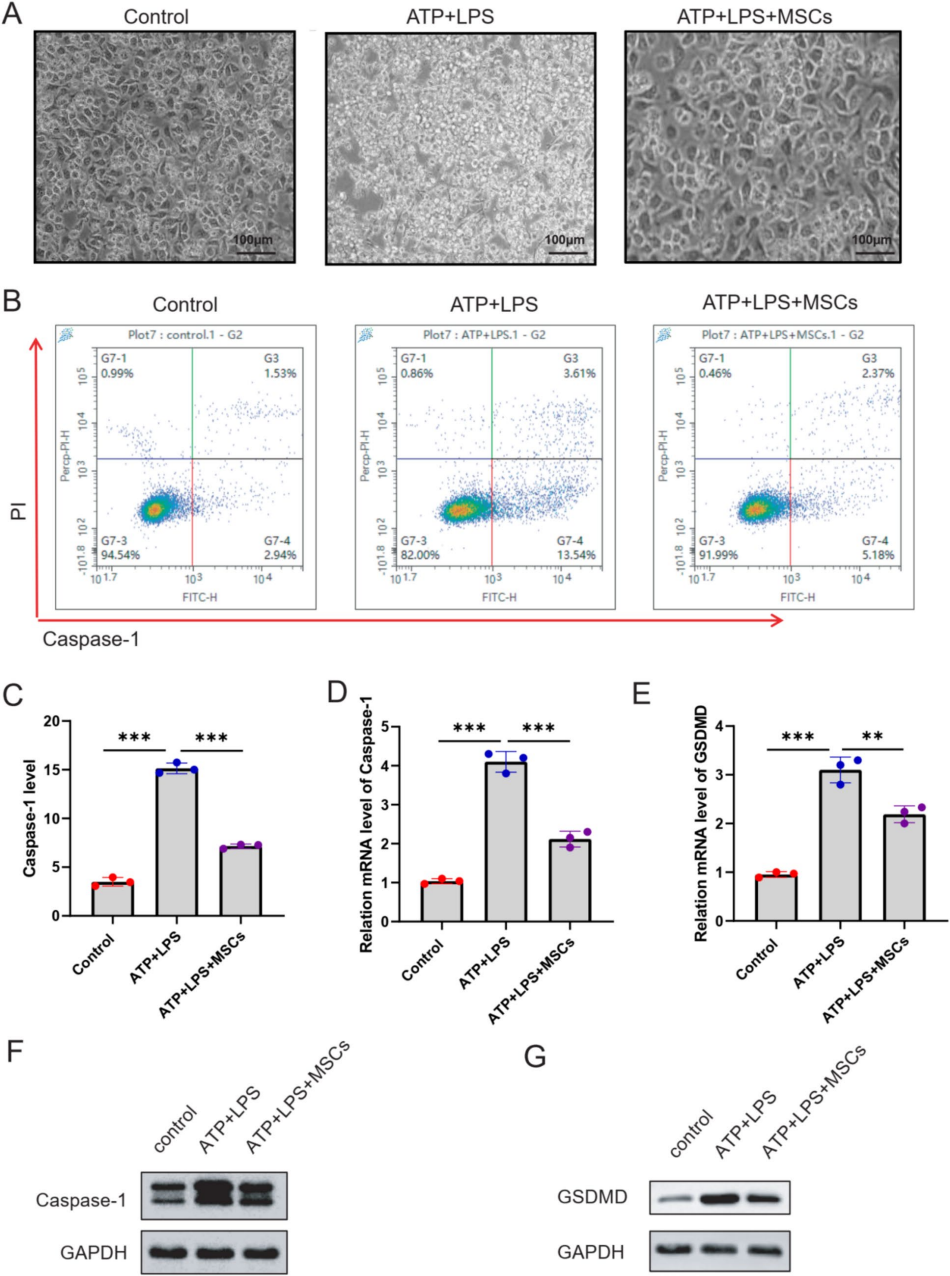
HucMSCs can inhibit hepatocyte pyroptosis. **A** The morphology of hepatocytes under the sub-microscope in correspondent groups (scale bar: 100 μm). **B**–**C** Cells with pyroptotic changes will show double positivity (the upper right quadrant) in both propidium iodide (PI) staining and caspase-1 activation. The ATP + LPS group showed more double-positive scatterplots than those in the ATP + LPS + MSC group. **D** RT-qPCR assay was used to detect the mRNA level of Caspase-1. **E** RT-qPCR assay was used to detect the mRNA level of GSDMD. The protein levels of caspase-1 (**F**) and GSDMD (**G**) were detected using WB assay. The experiment was performed by biological repeats. The data are represented by three individual experiments. *P < 0.05; **P < 0.01; ***P < 0.001

Next, the pyroptosis marker caspase-1 activity was detected using flow cytometry. The morphology of hepatocytes was examined using cell microscopy. Compared with the control group, hepatocytes in the ATP + LPS group exhibited significant swelling and enlargement, accompanied by the appearance of several bubble-like protrusions. In contrast, hepatocytes in the ATP + LPS + hucMSCs group maintained a normal polygonal shape (Fig. 2A). Particularly, flow cytometry analyses showed that ATP and LPS stimulation (14.91%) significantly increased the number of cells double-positive in caspase-1 and PI, compared with that of control groups (0.49%), while hucMSCs reduced the number of caspase-1 and PI positive cells (6.85%) (Fig. 2B–C), which indicated that hucMSCs treatment prominently reduced ATP- and LPS-induced hepatocyte pyroptosis. Gasdermin-D (GSDMD) is a key effector mediating macrophage pyroptosis downstream of the inflammasome. Next, the GSDMD mRNA level was detected by using RT-qPCR assay. RT-qPCR assay found that Caspase-1 and GSDMA mRNA level was increased in ATP + LPS group, hucMSCs decreased the mRNA levels of Caspase-1 and GSDMA in ATP + LPS-induced LO2 cells (Fig. 2D and E). Moreover, WB assay found that Caspase-1 and GSDMA protein level was increased in ATP + LPS group, hucMSCs decreased the protein levels of Caspase-1 and GSDMA in ATP + LPS-induced LO2 cells (Fig. 2F and G).

### Morphological identification of hucMSCs-Exos and its inhibitory effect on hepatocyte pyroptosis

**Fig. 3:**
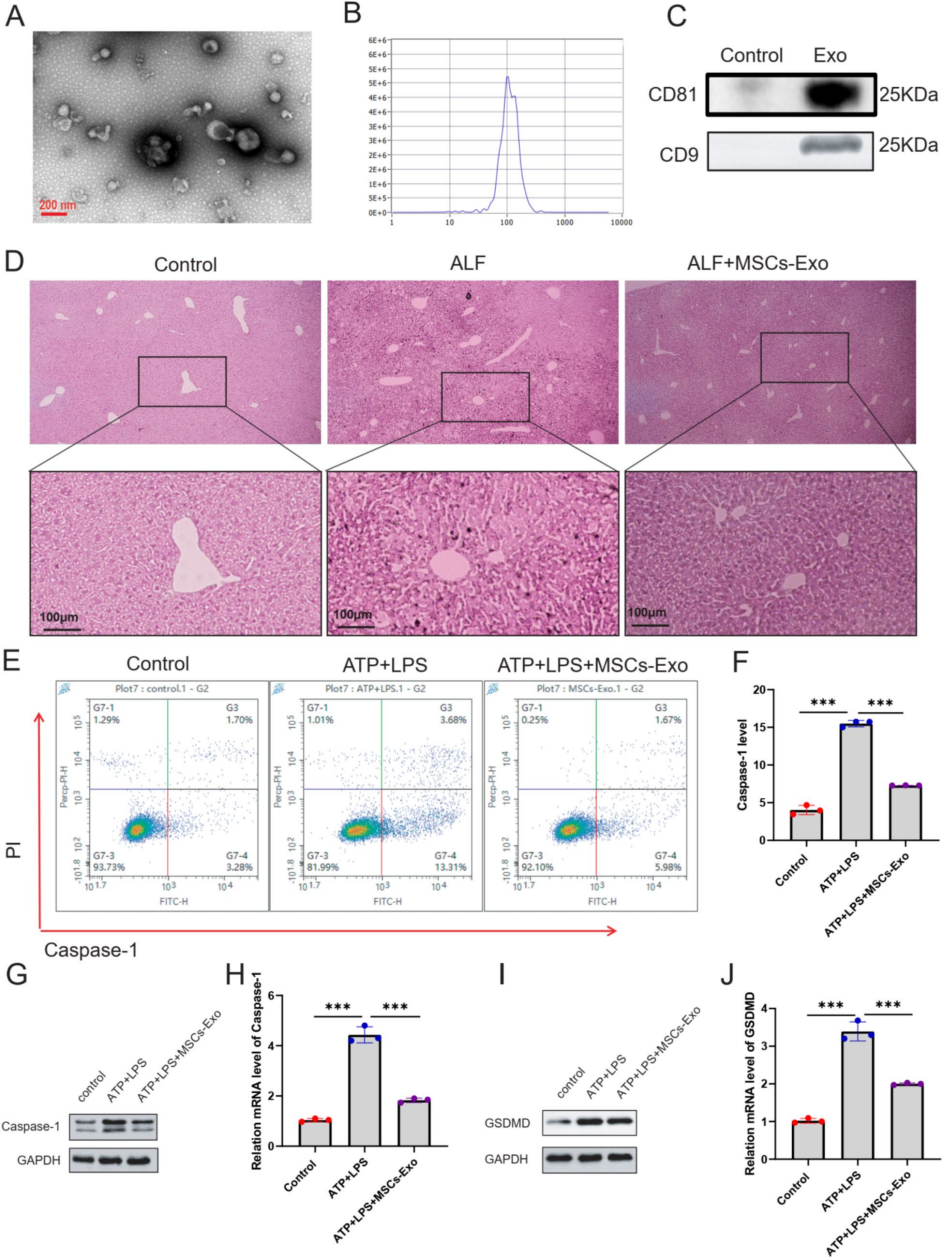
Morphological identification of hucMSCs-Exos and their pyroptosis-inhibitory effect on hepatocytes. **A** Morphology of hucMSCs-derived exosomes (hucMSCs-Exos) under transmission electron microscope (TEM) (scale bar: 200 nm). **B** Calculated size distribution of purified exosomes derived from hucMSCs culture medium by nanoparticle-tracking analysis (NTA). **C** Exosome marker proteins (CD9 and CD81) were detected by Western blotting. **D** H&E staining of liver tissues from ALF mice treated with hucMSCs-Exos (Scale bar: 100 μm). **E**–**F** The effect of hucMSCs-Exos on LO2 cell pyroptosis was evaluated using flow cytometry analysis with the FAM FLICA™ caspase-1 (YVAD) Assay kit. This kit specifically detects the active form of caspase-1, which is a key indicator of pyroptosis. The results showed that hucMSCs-Exos treatment significantly decreased the number of cells double-positive for active caspase-1 and PI. The mRNA level of caspase-1 (**G**) and GSDMD (**H**) were detected using RT-qPCR assay. The protein levels of caspase-1 (**I**) and GSDMD (**J**) were detected using Western blotting assay. The experiment was performed by biological repeats. The data are represented by three individual experiments. *P < 0.05; **P < 0.01; ***P < 0.001

Initially, exosomes derived from hucMSCs (hucMSCs-Exos) underwent a comprehensive characterization through transmission electron microscopy (TEM) (Fig. 3A), nanoparticle-tracking analysis (NTA), and western blot, aiming at having the appearance, morphology, and particle size characterized for further process. We found that the collected vesicles were consistent in size and morphology with the exosomes, showing a horseshoe shape with a diameter of nearly 50–100 nm (Fig. 3B). Furthermore, western blotting results validated the presence of exosomal surface markers CD9 and CD81 on the collected vesicles (Fig. 3C). The above findings showed that the obtained hucMSCs-Exos complied with the established standards. Next, the vivo model of ALF was established by the administration of APAP. H&E staining was used to assess the pathological changes. Post-APAP injection, inflammatory cells were evident in the liver, which were notably reduced with the administration of hucMSCs-Exo, indicating improved ALF outcomes (Fig. 3D). We verified the potential pyroptosis-inhibitory function of hucMSCs-Exos by treating the cells with (ATP+LPS+Exo) and without (ATP+LPS) hucMSCs-Exos, dividing samples into three groups, and evaluated whether exosomes functioned against pyroptosis by detecting the number of caspase-1 and PI double-positive cells (Fig. 3E and F). The effect of hucMSCs-Exos on LO2 cell pyroptosis was evaluated using flow cytometry analysis with the FAM FLICA™ caspase-1 (YVAD) Assay kit. This kit specifically detects the active form of caspase-1, which is a key indicator of pyroptosis. Flow cytometry analysis showed that the number of caspase-1 and PI double-positive cells was considerably increased in the ATP+LPS group (16.43%), compared to the untreated control group (0.62%), indicating that the pyroptotic cell model was successful. Meanwhile, the number of caspase-1 and PI double-positive cells in the ATP+LPS+Exos group (5.93%) was markedly lower (*p*<0.05) than the ATP+LPS group, implied the essential role hucMSCs-Exos played in regulating hepatocyte pyroptosis by reducing the number of damaged cells. Further, RT-qPCR assay revealed that hucMSCs inhibited the mRNA level of Caspase-1 and GSDMD in ATP+LPS-induced LO2 cells (Fig. 3G and H). Moreover, WB assay revealed that hucMSCs-Exo inhibited the protein level of Caspase-1 and GSDMD in ATP+LPS-induced LO2 cells (Fig. 3I and J).

### hucMSCs-exosomal miR-423-5p inhibited hepatocyte pyroptosis in ALF

**Fig. 4:**
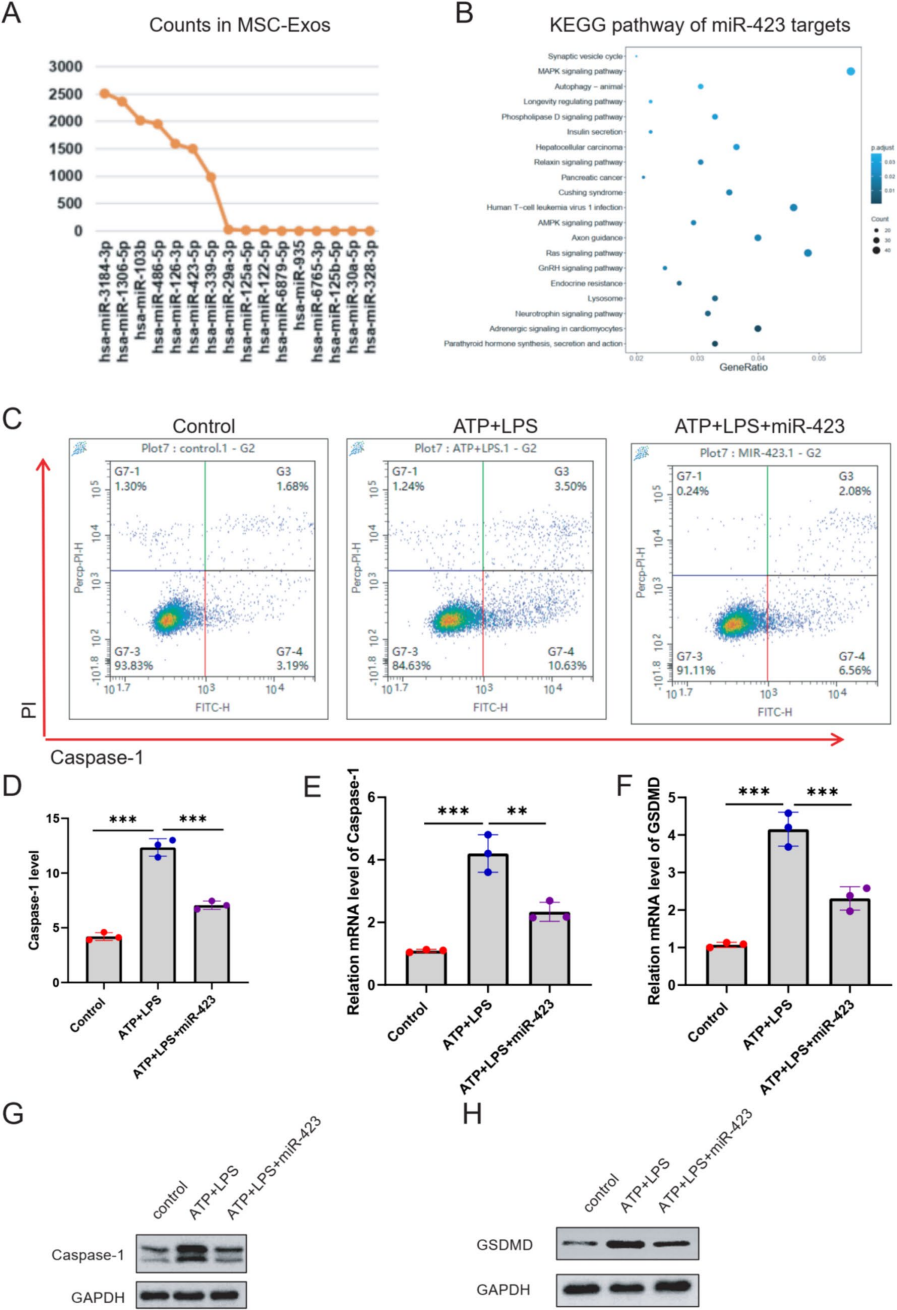
Identification of hucMSC-derived exosomal miRNA involved in hepatocyte pyroptosis. **A** MiRNA-seq was performed using hepatocytes from ATP + LPS and ATP + LPS + Exo group to find the differently expressed miRNAs. **B** The relative role of miR-423-5p in different signal pathways by bioinformation analysis. **C**–**D** The effect of miR-423-5p on LO2 cell pyroptosis was evaluated using flow cytometry analysis, showing that miR-423-5p treatment significantly decreased the number of caspase-1 and PI double-positive cells. The mRNA level of caspase-1 (**E**) and GSDMD (**F**) were detected using RT-qPCR assay. The protein level of caspase-1 (**G**) and GSDMD (**H**) were detected using WB assay. The experiment was performed by biological repeats. The data are represented by three individual experiments. *P < 0.05; **P < 0.01; ***P < 0.001

To establish the involvement of hucMSCs-derived exosomal miRNAs in hepatocytes pyroptosis, we identified their presence in the hucMSCs-Exos by performing miRNA sequencing (miRNA-Seq). As a result, miR-3184-3p, miR-1306-5p, miR-103b, miR-486-5p, miR-126-3p, miR-423-5p, and miR-339-5p were significantly up-regulated in hucMSCs-Exos (Fig. 4A). Subsequently, we delved into identifying the target genes of these miRNAs. Particularly, miR-423-5p emerged as a vital regulator in cell proliferation and death, playing a significant role in the RAS and MAPK signaling pathway (Fig. 4B), both of which are linked to the onset and progression of pyroptosis. We next explored the function of miR-423-5p on hepatocyte pyroptosis. Flow cytometry showed a significant decrease in the number of caspase-1 and the PI double-positive cells (4.85%) of ATP+LPS+miR-423-5p group, compared to ATP+LPS+NC group (14.26%, *p*<0.05) (Fig. 4C and D). Further, RT-qPCR assay revealed that miR-423 over-expression inhibited the mRNA level of Caspase-1 and GSDMD in ATP+LPS-induced LO2 cells (Fig. 4E and F). Additionally, a western blot assay demonstrated that miR-423 over-expression inhibited the protein level of Caspase-1 and GSDMD in ATP+LPS-induced LO2 cells (Fig. 4G and H).

### miR-423-5p targeted ZBP1 to induce hepatocyte pyroptosis

**Fig. 5:**
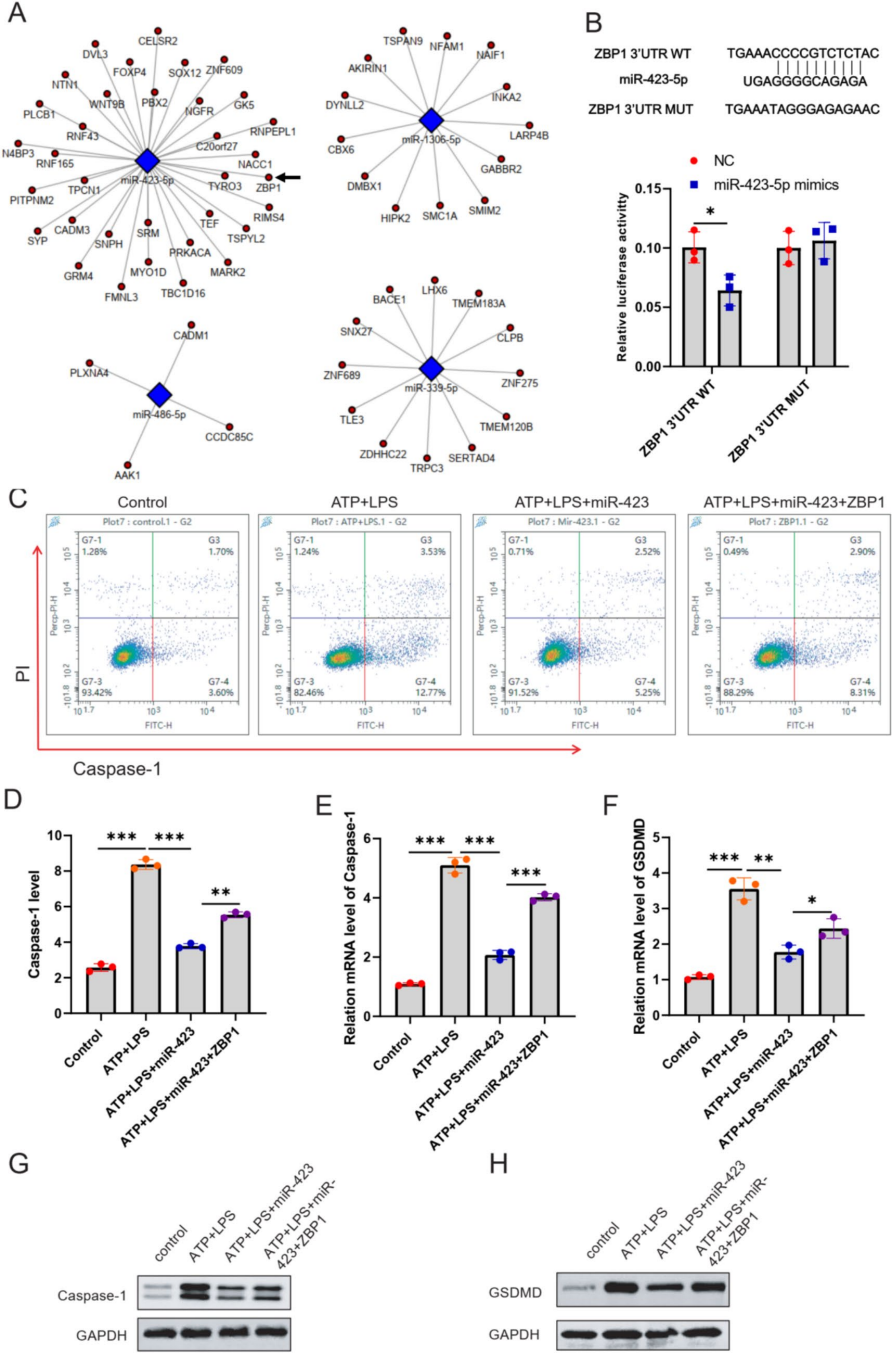
miR-423-5p inhibited hepatocyte pyroptosis by targeting ZBP1. **A** Target mRNAs of four sequenced miRNAs (miR-423-5p, miR-1306-5p, miR-486-5p, and miR-339-5p). Protein–Protein Interaction (PPI) networks were predicted using Targetscan and miRanda. **B** The binding sequences between miR-423-5p and ZBP1 3’-UTR. 293 T cells were transfected with a miR-423-5p mimic or Negative Control, together with wild type (WT) or 3’-UTR mutant type (MUT) from ZBP1 reporter vector. The relative luciferase activity was analyzed 48 h later. **C**–**D** The effect of miR-423-5p on LO2 cell pyroptosis was evaluated using flow cytometry analysis, showing that miR-423-5p treatment significantly decreased the number of caspase-1 and PI double-positive cells. The mRNA level of caspase-1 (**E**) and GSDMD (**F**) were detected using RT-qPCR assay. The protein level of caspase-1 (**G**) and GSDMD (**H**) were detected using WB assay. The experiment was performed by biological repeats. The data are represented by three individual experiments. *P* < 0.05 (*n* = 3)

miR-423-5p inhibited hepatocyte pyroptosis by targeting ZBP1. To gain initial insights into the understanding mechanism of miR-423-5p in regulating pyroptosis, we conducted for potential miR-423-5p targets. The protein–protein interaction (PPI) network diagram, which illustrates the downstream target proteins of the miRNAs obtained in section 4.4 (Fig. 5A), highlighted Z-DNA-binding protein 1 (ZBP1) as our focal point. Further, we confirmed the predicted binding sequence between miR-423-5p and 3’-UTR of WT ZBP1 using Luciferase reporter gene experiment (Fig. 5B). Based on this verification, we synthesized miR-423-5p mimics and negative control, transfected them into 293 T cells, and further confirmed the interaction between miR-423-5p and ZBP1 3’-UTR by luciferase reporter assay. Moreover, ZBP1 over-expression significantly induced hepatocyte pyroptosis in ATP+LPS-induced LO2 cells (Fig. 5C and D). ZBP1 up-regulated the mRNA level of caspase-1 and GSDMD in ATP+LPS-induced LO2 cells (Fig. 5E and F). Strikingly, ZBP1 over-expression restored the role of miR-423-5p reduced the diminished pyroptosis role of miR-423-5p in the ATP and LPS-induced LO2 cells.

## Discussion

Our study elucidates the novel role of hucMSCs-derived exosomal miR-423-5p in modulating hepatocyte pyroptosis, a critical factor in the pathology of ALF. By focusing on the microRNA content of hucMSC-derived exosomes, particularly miR-423-5p, we have identified a pivotal mechanism by which these exosomes inhibit pyroptosis in hepatocytes. This discovery offers a promising therapeutic avenue for the treatment of ALF.

Previous research has primarily focused on the therapeutic potentials of MSCs themselves in ALF, with multiple investigations showcasing the advantages of MSC transplantation in improving liver function and reducing inflammation. Initial reports suggested the potential contribution of hucMSCs to liver repair by differentiating into hepatocyte-like cells both *in vivo* and *in vitro* [39]. However, subsequent studies challenged this notion, indicating that the number of hucMSCs-derived cells detected *in vivo* after transplantation was insufficient to replace damaged hepatocytes and restore liver function [40, 41]. Recently, immunoregulation has emerged as a novel mechanism through which hucMSCs might exert therapeutic effects [42]. Subsequent studies confirmed that hucMSCs could participate in liver repair by promoting cell proliferation and suppressing cell death [43], which is a benefit for using hucMSCs to alleviate ALF. Our findings build on this foundation but diverge significantly by demonstrating that the exosomes derived from hucMSCs, independent of the cells, can confer significant hepatoprotective effects. This shift from cellular to acellular therapies in our study highlights a significant innovation, reducing potential risks associated with stromal cell therapies, such as immune rejection or tumor formation.

Our results demonstrated that hucMSC-derived exosomes significantly decrease the activation of caspase-1 and the occurrence of pyroptosis in hepatocytes, as shown in both in vitro and in vivo models. This finding aligns with our first research question regarding the mechanism by which hucMSC-derived exosomes exert their hepatoprotective effects.

Pyroptosis, a form of programmed lytic cell death in hepatocytes, is recognized as a significant pathological feature in ALF [44]. However, the precise mechanism underlying the function of hepatocyte pyroptosis in ALF remains to be fully elucidated. In this study, we initially utilized the liver cell line LO2, treated with ATP and LPS, to establish an ALF model. Through identifying positive signals for both caspase-1 activation and propidium iodide (PI) staining, we observed the activation of hepatocyte pyroptosis during the progression of ALF. As LPS is a major component of Gram-negative bacteria and is known to induce abnormal production of pro-inflammatory cytokines, its presence in the ALF model contributes to the distinctive pyroptotic changes characterized by excessive inflammatory responses and hepatocyte pyroptosis [45, 46]. Co-stimulation with LPS and ATP on hepatocytes creates cell models that closely resemble ALF, exhibiting pronounced inflammatory responses and hepatocyte pyroptosis [41]. Previous studies have demonstrated that inhibiting LPS/ATP-induced NLRP3-mediated pyroptosis can attenuate liver injury [47]. Collectively, these findings underscore the pivotal role of hepatocyte pyroptosis in the pathogenesis of ALF, providing a novel target for potential ALF treatments.

Recently, hucMSCs have been explored as a potential therapeutic option for diseases, including ALF. However, the underlying mechanisms by which hucMSCs exert their therapeutic effects remain poorly understood. MiRNAs are known to be transported through exosomes and play a crucial role in a variety of cellular activities [48]. Recent studies have increasingly highlighted the importance of exosomal proteins and microRNAs (miRNAs) derived from hucMSCs in regulating cellular physiological activities and modifying the microenvironment in target cells, which are crucial events in liver pathology [49]. Based on the existing findings, we hypothesized that hucMSCs-derived exosomal miRNAs may be key players in inhibiting pyroptosis and repairing liver damage. To identify the target genes of hucMSCs-Exos, we conducted miRNA sequencing and predicted target genes. We further analyzed the target genes of seven significantly up-regulated miRNAs through GO analysis and KEGG pathway enrichment analysis. Among these miRNAs, only the target genes of miR-423-5p were significantly enriched in pathways related to inflammatory and pyroptosis.

MicroRNA-423-5p (miR-423-5p) has been implicated in various diseases, where it plays a regulatory role in cell proliferation and apoptosis. It has also emerged as a potential biomarker for inflammatory conditions [50–52]. Recent studies have identified NLRP3 as one of the targets of miR-423-5p [53]. Consequently, we hypothesized that miR-423-5p may regulate pyroptosis, providing a plausible explanation for its impact on the development of acute liver failure (ALF). However, the specific relationship between miR-423-5p and ALF remains to be fully unclear. To address this, we extracted exosomal miR-423-5p and confirmed its substantial inhibitory effect on hepatocyte pyroptosis in an ALF cell model. Nevertheless, the detailed and specific mechanisms underlying the function of miR-423-5p in the progression of pyroptosis warrant further exploration. This is a convincingly proof indicating the pyroptosis-inhibitory effect that hucMSCs-derived miR-423-5p has on hepatocytes.

We identified miR-423-5p as a crucial mediator in this process, targeting ZBP1, a protein significantly involved in the pyroptotic pathway. This direct targeting not only clarifies the molecular pathway involved but also positions miR-423-5p as a potential therapeutic target. ZBP1, identified as one of the potential targets, is a crucial protein associated with pyroptosis and implicated in various human inflammatory diseases [54]. Notably, ZBP1 over-expression significantly enhances caspase-1 activation, a recognized marker of pyroptosis. While ZBP1’s role in promoting cell pyroptosis has been reported in acute pancreatitis mouse models, its correlation with hepatocyte pyroptosis in ALF has not been explored until now. Our study confirmed the presence and involvement of ZBP1 in the pathogenesis of ALF. Furthermore, the luciferase assay results provided additional verification of the binding site between ZBP1 and miR-423-5p. In summary, our research, employing exosomal miR-423-5p, successfully inhibited pyroptosis in LO2 liver cells, leading to reduced hepatocyte injuries in the context of ALF. However, for a comprehensive understanding, further evidence and a detailed elucidation of the relationship between miR-423-5p and ZBP1, and the precise mechanisms influencing pyroptosis, warrant further exploration.

Our study highlights the potential of leveraging exosome-mediated delivery of specific miRNAs as a strategy for cell-free therapy in ALF. The reliability of our findings is supported by the use of both technical and biological repeats in all experiments, ensuring the robustness of our results. Further investigations are warranted to assess the therapeutic efficacy and safety of synthetically engineered exosomes packed with specific miRNAs like miR-423-5p. This approach has the potential to enhance the precision and efficiency of treatments for ALF.

## Conclusion

In conclusion, our study provides the first evidence that exosomal miR-423-5p derived from hucMSCs can effectively inhibit pyroptosis in LO2 cells induced by ATP and LPS. Moreover, we demonstrated that hucMSCs-derived exosomal miR-423-5p exerts its effects by directly binding to the 3'UTR of ZBP1. Our study not only advances the understanding of the protective mechanisms of hucMSCs-derived exosomes in ALF but also opens new avenues for the development of targeted, cell-free therapies. This approach mitigates some of the limitations and risks associated with direct stromal cell therapies, marking a significant step forward in the treatment of liver diseases.

## Supplementary Information

Below is the link to the electronic supplementary material.

Supplementary file1 (DOCX 155 KB)

## Data Availability

miRNA-sequencing data are deposited on the National Centre for Biotechnology Information (http://www.ncbi.nih.gov/) GenBank database as individual BioProjects (PRJNA1148293).
