# Supplementary material for: Exosomes derived from human umbilical cord mesenchymal stem cells inhibit hepatocyte pyroptosis via miR-423-5p/ZBP1 in acute liver failure
Source: Hum Cell. 2025 Jul 4;38(5):124. doi: 10.1007/s13577-025-01248-1 (PMC12227468; doi:10.1007/s13577-025-01248-1)
Supplement: Supplementary file 1 — Supplementary file1 (DOCX 155 KB) [file 13577_2025_1248_MOESM1_ESM.docx]

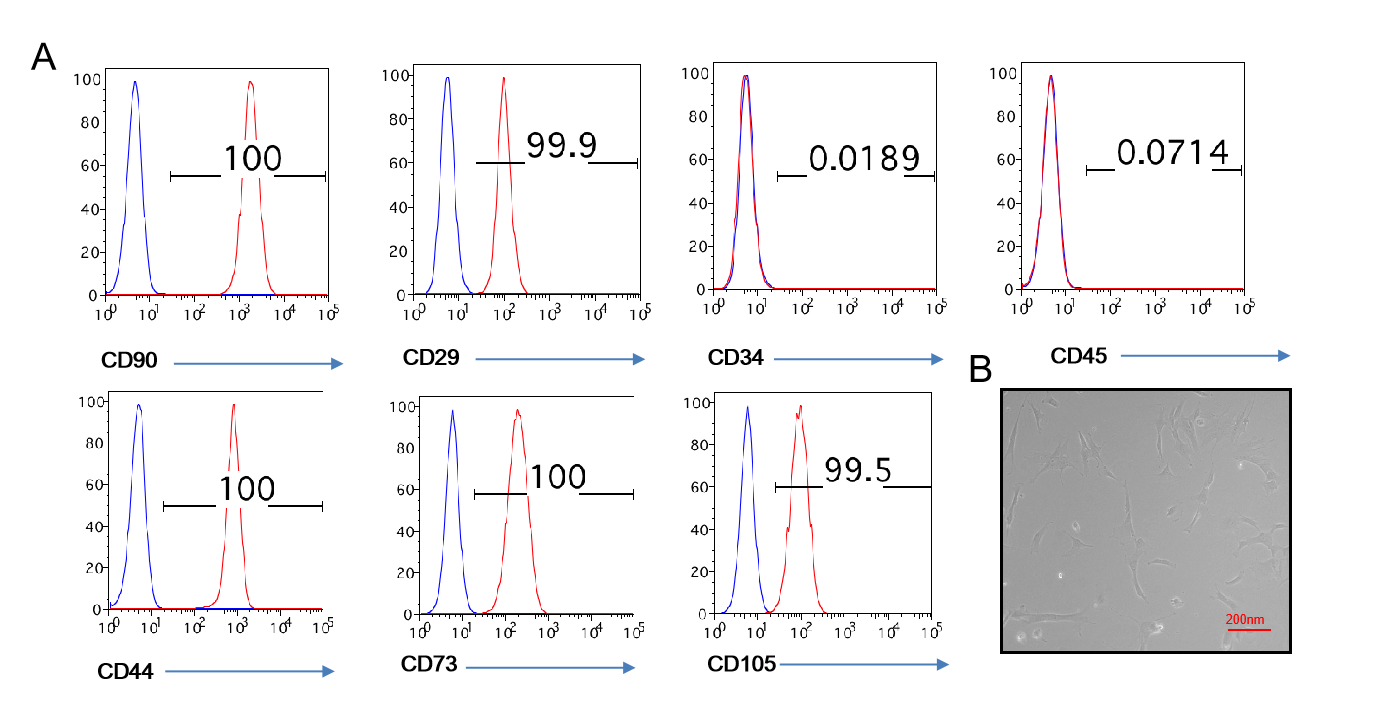


**Supplementary Figure 1** Immunophenotype identification and morphological observation of the hucMSCs involved in this study. (A) The immunophenotype of hucMSCs through flow cytometry, showing positivity in CD90, CD29, CD44, CD73, and CD105, and negativity in CD34, CD45. (B) All involved hucMSCs were examined using an Optical Microscope, confirming their authenticity and bioactivity. (Scale bar: 200nm). The experiment was performed by biological repeats.
